# Evaluation of a fermented palm kernel meal as a prebiotic for enhancing immune response in Indonesian red claw crayfish (*Cherax quadricarinatus*)

**DOI:** 10.14202/vetworld.2025.896-906

**Published:** 2025-04-19

**Authors:** Diki Danar Tri Winanti, Hilma Nahwa Firdausi, Maulid Wahid Yusup, Putu Cinthia Delis, Agus Setyawan, Yeni Elisdiana, Hilma Putri Fidyandini, Muhammad Kholiqul Amiin, Ari Widodo

**Affiliations:** 1 Department of Agriculture Product Technology, Faculty of Agriculture, Universitas Lampung, Lampung, 35141, Indonesia; 2 Department of Aquaculture, Faculty of Agriculture, Universitas Lampung, Lampung, 35141, Indonesia; 3 Department of Aquatic Resources, Faculty of Agriculture, Universitas Lampung, Lampung, 35141, Indonesia; 4 Department of Marine Science, Faculty of Agriculture, Universitas Lampung, Lampung, 35141, Indonesia; 5 Department of Aquaculture, National Taiwan Ocean University, Keelung, 20224, Taiwan, Republic of China; 6 Department of Biology, Faculty of Mathematics and Natural Science, Indonesia Defense University, Bogor, 16810, Indonesia

**Keywords:** aquaculture, crustacea, immune systems, palm kernel meal, prebiotic

## Abstract

**Background and Aim:**

The Indonesian red claw crayfish (*Cherax quadricarinatus*) represents a significant aquaculture commodity with substantial economic importance; however, disease outbreaks, particularly tail ulceration caused by *Aeromonas hydrophila*, pose challenges to sustainable production. This study evaluated the efficacy of fermented palm kernel meal (FPKM), rich in mannan oligosaccharides, as a prebiotic additive to enhance non-specific immune responses in *C. quadricarinatus*.

**Materials and Methods:**

A total of 120 crayfish (mean weight 10.3 ± 0.15 g and length 7.53 ± 0.26 cm) were allocated in a completely randomized design comprising four dietary treatments: a control group without FPKM and probiotics and three experimental groups receiving diets supplemented with FPKM at concentrations of 40, 80, and 120 g/kg along with probiotics. Hemolymph samples were collected at baseline (day 0) and on days 3, 5, 7, and 14 post-treatment. Non-specific immune responses evaluated included total hemocyte count (THC), differential hemocyte count (DHC), phagocytosis activity (PA), phagocytosis index (PI), phenol oxidase (PO) activity, superoxide dismutase (SOD) activity, total plasma protein (TPP), and immune gene expression (lipopolysaccharide and β-1,3-glucan-binding protein [LGBP], lectin).

**Results:**

The inclusion of FPKM significantly increased THC, with peak enhancement observed on day 7 at the highest FPKM concentration (120 g/kg). DHC remained stable across treatments. Phagocytic parameters, including PA and PI, showed significant improvements (74.5 ± 12.5% and 2.8 ± 0.41, respectively) by day 7 in the 120 g/kg treatment. PO and SOD activities significantly increased on days 5 and 14, respectively, with optimal responses at the highest dietary FPKM inclusion. TPP levels did not exhibit significant variation among treatments. Molecular analyses revealed marked upregulation of immune-related genes, notably lectin and LGBP, with peak expression detected in the 40 g/kg FPKM group.

**Conclusion:**

Supplementing diets with FPKM substantially improved non-specific immune responses in *C. quadricarinatus*. Optimal immune enhancement was generally achieved with 120 g/kg FPKM inclusion, although significant molecular immune responses were evident at lower concentrations. The findings underscore the potential of FPKM as a natural prebiotic to sustainably enhance crayfish immunity, thereby reducing dependency on antibiotics and contributing to eco-friendly aquaculture practices.

## INTRODUCTION

*Cherax quadricarinatus* is an important fishery commodity in Indonesia and worldwide. It has great potential to be cultured because consumers highly prefer it. The demand for *C. quadricarinatus* is high in domestic and international markets. Based on the Badan Pusat Statistik (Statistics Indonesia), in 2017–2022, Indonesia’s red claw crayfish export volume reached an average of 1.85 thousand tons/year. However, its production cannot meet the high market demand. This can occur because of several problems related to aquaculture activities. A problem in the culture of crayfish is disease infection. Disease infection can occur due to environmental factors, such as high stocking density, which leads to poor water quality management. Some diseases that frequently infect crayfish are *Macro-brachium rosenbergii* nodavirus [1], yellow head virus, *C. quadricarinatus* parvo-like virus, white spot syndrome virus, and tail blister disease caused by *A.hydrophila* [2]. The prevention of disease in crayfish is achieved by boosting the immune system. Enhancing the immune system can be achieved by adding prebiotics to the diet. Non-digestible food ingredients that cooperate with beneficial microbes (probiotics) in digestion are commonly defined as prebiotics [3]. Prebiotics are widely used to improve growth and the fish’s immune system [4]. Prebiotics are symbiotic with microflora in the gut, eliminating pathogenic bacteria in the digestive tract [5]. The many types of prebiotics that have been applied are fructose oligosaccharides (FOS), mannan oligosaccharides (MOS), inulin, and β-glucan [6].

Fermented palm kernel meal (FPKM) is a source of MOS that can be applied to crayfish. The fermentation process in palm kernel meal (PKM) can digest mannan and produce enzymes. Enzymatic digestion converts mannan into MOS [7]. A study conducted by Awasthi *et al*. [6] proved that it is effective to use MOS prebiotics derived from *Saccharomyces cerevisiae* cell membranes to enhance the health status and increase the ability to treat pathogens and stress conditions in crayfish. The fermentation of PKM is essential because of its high crude fiber content. High fiber content makes it difficult to digest because limited enzymes can hydrolyze crude fiber in fish or crustacean digests [8]. An attempt to reduce the crude fiber content in PKMs is to ferment it with the aid of the mold *Aspergillus niger*. Based on the results of preliminary studies conducted, fermentation using *A. niger* can reduce total crude fiber by 21% and increase crude protein by 86%. Based on these results, MOS derived from FPKM has great potential as a prebiotic in feed.

Synbiotics, which combine prebiotics and probiotics, have shown superior results compared to individual components [9]. Various prebiotic-probiotic combinations have been tested, including MOS and xylooligosaccharide with *Enterococcus faecalis* and *Pediococcus acidilactici* [10], galactooligosaccharide with *E. faecalis* [11], and olive leaf extract with *Lactobacillus reuteri* and *Bacillus clausii* [12]. These synbiotic diets have improved growth rates, survival, immune responses, and resistance to pathogens like *Aeromonas hydrophila*. Synbiotics enhance antibacterial activities in shell mucus, increase beneficial gut bacteria, and improve nutrient digestibility [9, 11]. The effectiveness of synbiotics appears to be dose-dependent, with optimal concentrations varying based on the specific combinations used [12].

Despite substantial advancements in crustacean aquaculture, significant challenges persist in maintaining the health and productivity of Indonesian red claw crayfish *(C. quadricarinatus*), primarily due to recurrent disease outbreaks such as tail ulceration caused by *A. hydrophila*. While prebiotics have demonstrated efficacy in enhancing immune responses in aquatic organisms, there remains a paucity of research investi-gating the immunomodulatory potential of locally available agricultural by-products, such as FPKM, particularly in crayfish. The literature currently lacks comprehensive assessments of the non-specific immune mechanisms and molecular immune responses elicited by FPKM-derived prebiotics in *C. quadricarinatus*.

Therefore, this study aimed to evaluate the effectiveness of FPKM as a prebiotic supplement in enhancing non-specific immune responses and immune-related gene expression in Indonesian red claw crayfish *(C. quadricarinatus*). The research specifically focused on determining optimal dietary inclusion levels of FPKM that maximally stimulates immune functions, thereby contributing to the development of sustainable, cost-effective, and antibiotic-free strategies to improve crayfish health and productivity in aquaculture systems.

## MATERIALS AND METHODS

### Ethical approval

All experiments in this study were approved by the Ethics Committee on the Use of Animals of the Federal University of Lampung under protocol (#UNILA-10/2023) and carried out according to the Guidelines and Regulations for Fish Experimentation at UNILA.

### Study period and location

The study was conducted from October 2022 to December 2023 at the Aquaculture Laboratory of the Department of Fisheries and Marine, Faculty of Agriculture, University of Lampung, Bandar Lampung, Lampung Province, Indonesia (5°21’56.2” S 105°14’33.1” E).

### Tools and materials

This study uses crayfish that were weighed (10.3 ± 0.15 g) and measured in total length (15.26 ± 0.24 cm). The facilities in this study used 60× 40 × 40 cm^3^ aquariums and were distributed in a completely randomized design with four treatments and three repetitions with details of P0: Feed without FPKM substitution and without probiotics, P1: Feed with 40 g/kg FPKM substitution and probiotics addition, P2: Feed with 80 g/kg FPKM substitution and probiotic addition, and P3: Feed with 120 g/kg feed FPKM substitution and probiotic addition. The equipment used in this study included a 1 ml syringe, Eppendorf tube, ethylenediaminetetraacetic acid tube, hemacytometer, microscope, micropipette, and centri-fugation. The materials used in this study were 70% alcohol, commercial probiotics, phosphate-buffered saline solution, distilled water, Giemsa, methanol, and Bradford reagent.

The fermentation of PKM was based on a previously reported procedure by Puastuti *et al*. [13] and has been modified. One kilogram of mashed PKM was mixed with 600 mL of water containing a mineral solution containing 1% ZA and 0.5% urea to support microbial growth. The mixture was then sterilized in an autoclave for 30 min. Next, it was maintained at room temperature until it had slightly cooled. Then, it was mixed with a starter of *A. niger* mold at up to 8 g/kg. The blended PKM was then placed in a pan and stored at room temperature (30°C) aerobically for 3–5 days. Finally, it was dried in an oven for 2 h at 35°C–40°C.

### Research procedure

The initial step in feed processing involves preparing the ingredients, including fish, soybean, corn, pollard, PKM, and premixed meal. All ingredients were then mixed and molded using a pellet molding machine. After molding, the pellets were dried in an oven. Then, proximate analysis was completed before it was given to the crayfish (Table 1). Before the feed was given to the crayfish, probiotics were added using the spray method. Feeding was conducted twice a day (08.00 and 17.00), using the maximum feeding rate based on the average weight of the crayfish to avoid overfeeding and compromising water quality. Feeding rates were updated as described by Trisnasari *et al*. [14]. The aquariums were cleaned every morning by siphoning, which was performed 30 min after the final feeding of the previous day. After cleaning the tanks, the water was replaced directly in the filter to maintain the system’s water volume.

**Table 1:** The results of the proximate analysis.

No.	Sample	Moisture content (%)	Ash content (%)	Lipid (%)	Protein (%)	Crude fiber (%)
1	Control	5.72	9.64	10.46	34.03	1.77
2	FPKM 40 g/kg	5.72	9.69	10.09	34.69	2.55
3	FPKM 80 g/kg	5.07	9.68	10.30	34.14	1.96
4	FPKM 120 g/kg	5.58	9.69	10.30	34.46	1.14

FPKM=Fermented palm kernel meal

### Collected samples and biological indexes

Four crayfish per treatment and repetition were weighed and measured, and after being anesthetized by an ice cube (50 g) with a continuous aeration supply, hemolymphs were collected (1 mL/tail). Hemolymphs were collected on day 0 (before treatment feed), day 3, day 5, day 7, and day 14. Hemolymphs were taken from the base of crayfish pereopods. Subsequently, 0.8 mL of hemolymph solution was taken using a 1-mL syringe flushed with anticoagulant. The hemolymph was then stored in an Eppendorf tube and placed in a container treated with ice [15]. This study evaluated parameters such as total hemocyte count (THC), differential hemocyte count (DHC), phagocytosis activity (PA), phagocytosis index (PI), total plasma protein (TPP), superoxide dismutase (SOD) activity, phenol oxidase (PO) activity, and immune gene expression (lipopolysaccharide and β-1,3-glucan-binding protein [LGBP], lectin).

### THC

Fresh hemocytes (10 μL) were diluted in 20 μL phosphate-buffered saline (PBS) (1:3 dilution) and homogenized by pipetting. The total number of hemocytes was calculated using a hemacytometer (Neubauer, Germany) and examined under a microscope at 100× magnification. Cells were counted and the average was used as the total number of hemocytes [15].


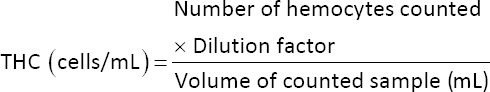


Example calculation: Number of hemocytes counted: 250 cells, dilution factor: 10, and volume of counted sample: 0.1 mL.


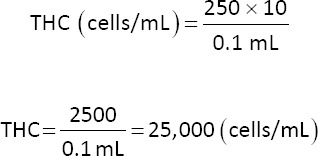


### DHC

The DHC was analyzed using the method described by Sang *et al*. [15] with some modifications. Fresh hemolymph (20 μL) was dripped onto glass, stained, and air-dried. The specimen was fixed in 70% methanol for 10 min. The smear preparations were stained with Giemsa (Merck, Germany) for 10 min. The cover glass was then put.


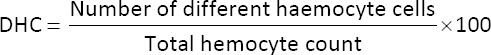


### PA and PI

The PA and PI were analyzed based on the study of Yudiati *et al*. [16] with some modifications. Fresh hemolymph (20 μL) was suspended in *Staphylococcus aureus*, formed into an object glass, and fixed in metha-nol. The specimens were stained with 10% Giemsa for 20 min. The formula for PA is as follows [17]:

PA = (a/b) × 100%

PI = c/a

Where, a = Number of phagocytic cells, b = Total number of cells examined, c = Number of bacteria phagocytosed. The preparations were then observed under a microscope with 100× magnification.

### PO activity

The PO activity was determined as described by Yudiati *et al*. [16]. Hemolymph (100 μL) was diluted with PBS (100 μL) (1:1 dilution) and then centrifuged at 700× *g* and 4°C for 20 min. The supernatant was discarded, and the sediments were added 100 μL cacodylate citrate buffer (0.1 M sodium cacodylate trihydrate; 0.45 M sodium chloride (NaCl), and 0.01 M sodium citrate), centrifuged at 700× *g*, at 4°C for 20 min. The supernatant was discarded, and 100 μL of cacodylate citrate buffer (0.01 M sodium cacodylate trihydrate; 0.45 M NaCl; 0.01 M CaCl_2_.2H_2_O; 0.26 M MgCl_2_.6H_2_O) was added to the sediments and inserted into a 96-well microplate. Subsequently, each well containing the sample was added with 100 μL trypsin (Sigma Aldrich), resuspended, and incubated for 10 min. Furthermore, 50 μL of levodopa was added, and the absorbance was measured using a microplate reader at a wavelength of 490 nm. The PO activity was assessed using the absor-bance values.

### SOD activity

The SOD activity was determined as described by Yudiati *et al*. [16]. A total of 50 μL of hemolymph were placed in a microtube, diluted with 150 μL of PBS (1:4 dilution), vortexed, and then centrifuged at 700× *g*. The supernatant was collected and heated in a water bath at 65°C for 5 min to obtain the crude SOD extract. Next, the supernatant was heated in a water bath at 65°C for 5 min to obtain the crude SOD extract. The SOD crude extract was stored at −20°C until use in the SOD assay. The SOD assay was conducted by taking 100 μL of SOD crude extract mixed with 50 μL of nitro blue tetrazolium reagent, NBT (NBT 0.1%), followed by incubation at room temperature for 2 min and measurement of absorbance at a wavelength of 600 nm was performed. SOD activity was assessed using these absorbance values.

### TPP

The TPP was analyzed according to a previously described procedure by Yudiati *et al*. [16]. A total of 15 μL of hemolymph were centrifuged at 700× *g* for 10 min. Then, 5 μL of supernatant was taken and poured into a 96-well microplate (Iwaki, Japan), and 250 μL of Bradford reagent (Bio-Rad, USA) was added and incubated for 10 min. The absorbance of each sample was then measured using a microplate reader (R-Bio pharm Well Reader, Germany) at a wavelength of 630 nm. A standard curve of protein content was prepared using bovine serum albumin (Merck) at different concentrations (0, 50, 100, 150, and 200 mg/mL).

### Immune gene expression

This study evaluated two immune genes: LGBP and lectin. Each gene was observed and compared with the housekeeping gene as an internal control. The primers used for each gene are listed in Table 2 [17, 18].

**Table 2:** Crayfish immune gene primers.

Gene	Primer	Sekuen (5’–3’)	Accession	References
LGBP	CqLGBP-qPCR-F	CAGCGGTGAGATTGACATT	KP100470	[17]
	CqLGBP-qPCR-R	TGGAAACTGTTAGCGAAGG		
Lectin	CqCTL-qRT-F	ATGGTGAAGGCATGTGTGACG	MN944107	[18]
	CqCTL-qRT-R	GCAGACCAAGGTCTCTTGCTCA		
β-actin (Internal control)	β-actin-F	ATCACTGCTCTGGCTCCTGCTACC		[17]
	β-actin-R	CGGACTCGTCGTACTCCTCCTTGG		

LGBP=Lipopolysaccharide and β-1,3-glucan-binding protein, qPCR=Quantitative polymerase chain reaction, F=Forward, R=Reverse

### Isolation of Hemolymph RNA

The RNA was isolated as described by Schmittgen and Livak [19]. 50 μL of hemolymph was added with 500 μL of Trizol solvent, homogenized using pipetting, and incubated at room temperature for 5 min. 100 μL of chloroform was added and stirred for 5 s and then incubated at room temperature for 3 min. Next, it was centrifuged at 12,000× *g* for 15 min. The supernatant was transferred to a 1.5 mL microtube. Furthermore, 250 μL of isopropanol was added and incubated for 15 min. The sample was centrifuged at 12,000× *g* for 10 min. Transfer the supernatant to a new 1.5 mL sterile microtube and add 1,000 μL of 75% ethanol. The samples were then centrifuged at 7,500× *g* for 5 min. The ethanol was removed using a micropipette. Finally, the pellets were dissolved with 50–100 μL of nuclease-free water and preserved at –20°C.

### Complementary DNA (cDNA) synthesis (reverse transcription)

The first method was to add 2 μL of RNA with a 100 ng/μL concentration and mixed thoroughly using a vortex. Subsequently, it was reacted at 42°C for 30 min. Next, the reverse transcriptase enzyme was inactivated at 94°C for 5 min. The composition of the materials used for cDNA synthesis is presented in Table 3.

**Table 3:** The materials for cDNA.

Materials	Volume (µL)
Buffer AMV	2.5
AMV transcriptase enzymes	0.125
Mix reverse primer	2
Nuclease free water	5.875
Total	10.5

AMV=Avian myeloblastosis virus, cDNA=Complementary DNA

### Quantitative polymerase chain reaction (qPCR)

Immune gene expression was analyzed using qPCR, following the standard protocol outlined by Setyawan [20]. LGBP and lectin were chosen as immune markers due to their crucial roles in the innate immune response of aquatic organisms. LGBP is essential for recognizing and binding pathogen-associated molecular patterns, such as bacterial lipopolysaccharides (LPS) and fungal β-glucans, triggering immune signaling pathways. Lectins play a key role in non-specific immunity by facili-tating pathogen recognition, agglutination, and immune cell activation. The materials and qPCR condi-tions are summarized in Tables 4 and 5.

**Table 4:** The materials for qPCR.

Materials	Volume (µL)
KAPA SYBR FAST qPCR kit master mix	6.25
Primer forward (10 µM)	0.25
Primer reverse (10 µM)	0.25
ROX Low reference dye	0.25
dH_2_O	10.5
Total	17.5

qPCR=Quantitative polymerase chain reaction

**Table 5:** The conditions for qPCR.

Program	Temperature (°C)	Time	Cycles
Pre-denaturation	94	5’	1
Denaturation	94	6”	
Annealing	55–60	18”	45
Extension	68	30”	
Final extension	68	7’	1
Melting curve	95	30’	

qPCR=Quantitative polymerase chain reaction

### Statistical analysis

The data obtained were statistically analyzed using analysis of variance (ANOVA), followed by Duncan’s multiple range test at a significance level of p < 0.05 for evaluating THC, DHC, PA, PI, TPP, lysozyme activity, SOD activity, and PO activity. Gene expression levels were quantified utilizing the comparative threshold cycle (CT) method described by Schmittgen and Livak [19], where relative expression was determined by fold-change values calculated with the formula 2^−ΔΔCT. Specifically, ΔΔCT was defined as ([CT_gene of interest−CT_internal control]_sample A−[CT_gene of interest−CT_internal control]_sample B). Fold-change values represented the relative alterations in crayfish immune gene expression. All experimental data were systematically organized using Microsoft Excel 2024 (Microsoft, Washington, USA) and statistically processed with the Statistical Package for the Social Sciences software version 26.0 (IBM Corp., NY, USA).

## RESULTS

### THC

**Figure 1:**
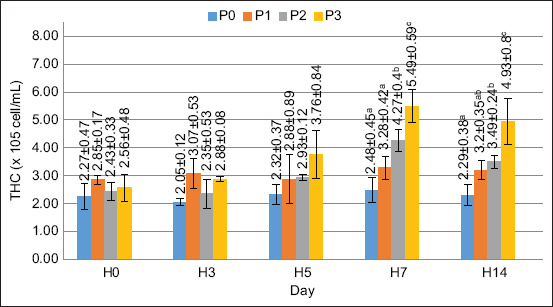
Total hemocyte count value. Remarks: Small letter notations at each treatment indicate significant differences (p < 0.05).

In the present study, the THC value was increased after supplementing the feed with PKM. The THC value of crayfish is presented in Figure 1.

Figure 1 illustrates the THC values over time in different treatment groups (P0, P1, P2, and P3). Initially, THC levels were relatively stable among all groups. However, from day 5 onward, a noticeable increase in THC was observed, particularly in P2 and P3, indicating an enhanced immune response. By day 7 and 14, THC levels were significantly higher at P2 and P3 than at P0 and P1, suggesting that these treatments positively influenced hemocyte proliferation. The best value for THC was obtained using P3, which is the application of FPKM (120 g/kg feed). Based on the results of the ANOVA, the application of FPKM did not significantly differ (p < 0.05) on days 0, 3, and 5, whereas the application of FPKM significantly differed in the THC value on days 7 and 14 (p < 0.05). This trend highlights the potential immune-boosting effects of dietary interventions in enhancing non-specific immunity.

### DHC

**Figure 2:**
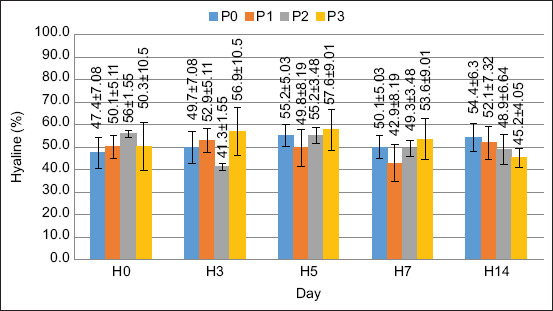
Hyaline cell density in crayfish (*Cherax quadricarinatus*).

**Figure 3:**
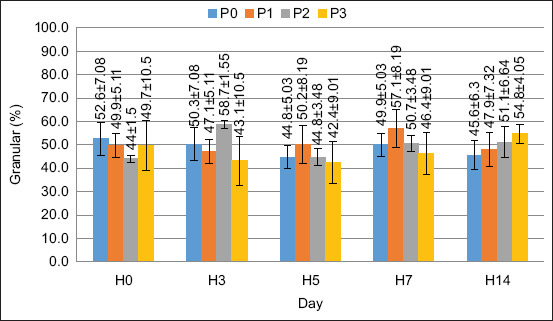
Granular cell counts of crayfish (*Cherax quadricarinatus*).

In this study, two cell types were observed: Hyaline and granular. The average DHC values are shown in Figures 2 and 3.

The DHC value was stable pre- to post-treatment. There was no significant increase in either hyaline or granular cell values. Based on the results of the ANOVA, FPKM supplementation in the feed had no significant effect on hyaline and granular cell values (p > 0.05).

### PA and PI

**Figure 4:**
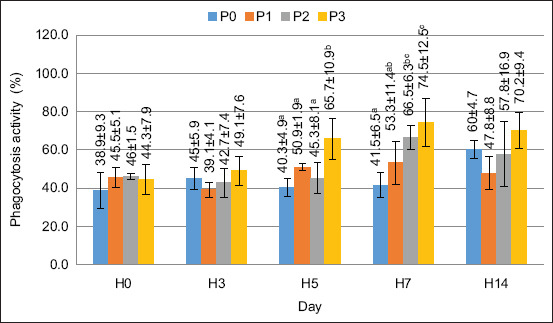
Phagocytosis activity value in crayfish (*Cherax quaricarinatus*) Remarks: Small letter notations at each treatment indicate significant differences (p < 0.05).

The value of PA in this study increased after supplementation with FPKM in the feed. Based on the ANOVA test, the supplementation of FPKM in feed had a significantly different effect on the PA on days 5 and 7 (p < 0.05). The PA value is shown in Figure 4.

**Figure 5:**
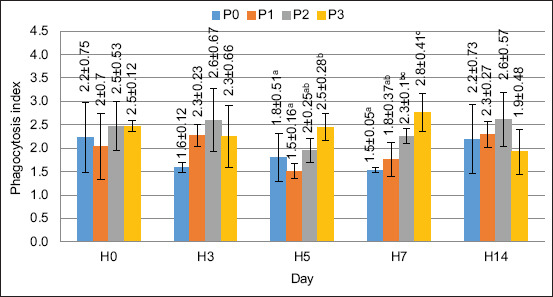
Phagocytosis index value in crayfish (*Cherax quaricarinatus*) Remarks: Small letter notations at each treatment indicate significant differences (p < 0.05).

The PI was measured along with PA. The PI was determined by determining the number of bacteria ingested by the phagocytic cells. The PI value is shown in Figure 5. The ANOVA results indicated that FPKM supplementation in the feed significantly affected the PI on days 5 and 7 (p < 0.05). The PI value in this study showed similar results to the PA value, which means that supplementing with FPKM substitution at up to 120 g/kg of feed gave the best results in increasing the PI value.

### PO activity

**Figure 6:**
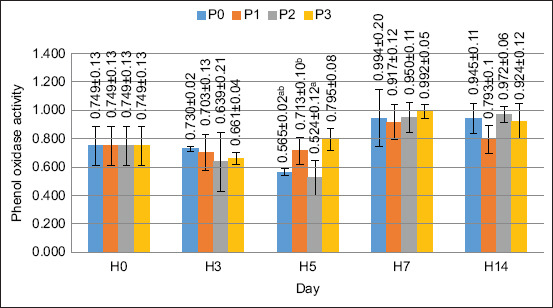
Phenol oxidase levels in crayfish (*Cherax quaricarinatus*) Remarks: Small letter notations at each treatment indicate significant differences (p < 0.05).

The results of the ANOVA test showed that the supplementation of PKM in feed gave significantly different results on the value of PO activity on day 5 (p < 0.05). Treatment with the addition of PKM at a concentration of as much as 120 g/kg of feed gave the best effect compared with the other treatments. The PO activity value is shown in Figure 6.

### SOD activity

**Figure 7:**
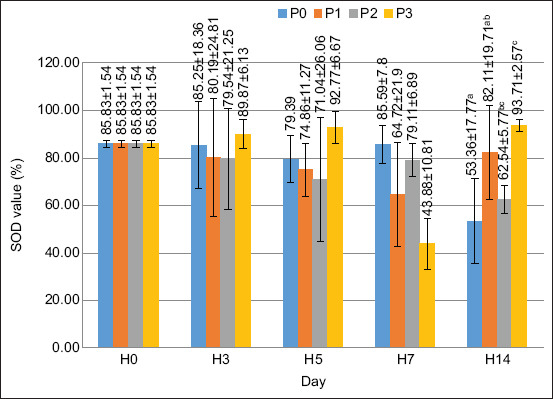
SOD value in crayfish (*Cherax quaricarinatus*) Remarks: Small letter notations at each treatment indicate significant differences (p < 0.05).

Based on the results of the ANOVA test, the supplementation of PKM in the feed had a significantly different effect on the SOD value on day 14 (p < 0.05), while on days 0, 3, 5, and 7, the results of the ANOVA test did not give a significantly different effect (p > 0.05). The SOD value is shown in Figure 7.

### TPP

**Figure 8:**
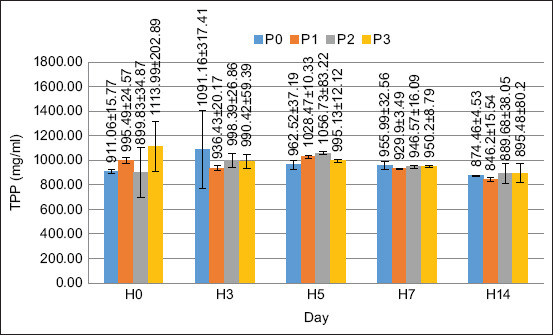
Total protein and plasma concentrations in crayfish (*Cherax quaricarinatus*).

In this study, the TPP value of each treatment did not significantly increase. TPP was observed on days 0, 3, 5, 7, and 14. The TPP values in all treatments tended to be in the same range at all observation times. Based on the results of the ANOVA, the supplementation of PKM in the feed did not have a significant effect on any of the treatments (p > 0.05). The TPP value in this study is shown in Figure 8.

### Gene expression

This study examined two immune genes, LGBP and lectin. This study uniquely demonstrates the upregulation of immune genes such as LGBP and lectin in crayfish-fed FPKM-supplemented diets. These findings provide novel insights into the field, as this is the first report of such molecular responses in crayfish or similar species. The lectin immune gene was highly expressed compared with the LGBP immune gene. The lectin immune gene exhibited the highest upregulation of 52,299.2-fold in P1 on day 7 and experienced the lowest down-regulation of −53.8-fold in P3 on day 3. The LGBP immune gene had the highest up-regulation (131.95-fold) in P1 on day 14 and the lowest down-regulation (17.47) in P1 on day 5. The results of the PCR analysis of immune genes are presented in Table 6.

**Table 6:** Gene expression values (fold change) in crayfish (*Cherax quadricarinatus*).

Treatment	Gene target	Day				

		0	3	5	7	14
FPKM (40 g/kg)	LGBP	−1.45	0.00	−17.47	66.94	131.95
	Lectin	−2.8	−6.7	−2.5	52,299.2	7,595.3
FPKM (80 g/kg)	LGBP	−4.06	1.52	0.00	−30.03	3.16
	Lectin	2.4	144.8	47.1	187.9	6.9
FPKM (120 g/kg)	LBGP	0.00	0.00	0.00	2.03	0.00
	Lectin	433.9	−53.8	51.0	−2.3	27,609.9

The minus mark (−) shows the down-regulation of gene expression. FPKM=Fermented palm kernel meal, LGBP=Lipopolysaccharide and β-1,3-glucan-binding protein

## DISCUSSION

Crayfish are crustaceans with unique defense systems that differ from those of finfish. Crayfish do not have a specific defense mechanism, and they only depend on the body’s non-specific (innate) defense system. The nonspecific immunity involves both cellular and humoral immune responses. In the cellular immune response, blood cells in glandular lymph are critical. Blood cells play a key role in defending the body against pathogens, including viruses, bacteria, fungi, protozoa, and metazoa [21]. Blood cells also promote the production of important molecules in the gland, such as adhesins, agglutinins, and antimicrobial peptides [22]. The body’s humoral defense system includes PO, prophenoloxidase (proPO), lectins, and agglutinins [23]. The primary immune process in crustaceans is the recognition of invading microorganisms through blood cells and plasma proteins [24].

Based on the hematological evaluation of this study, we found that substituting FPKM can increase the crayfish’s body defense system. It can be seen from the increase in several parameters after treatment, especially the activities of THC, PA, PI, SOD, and PO. The THC parameter, the number of blood cells in crayfish, increased during FPKM supplementation. Based on the ANOVA test results, there was a significant effect on THC values (p < 0.05). The higher THC on days 3, 5, and 7 was determined by the efficacy of MOS FPKM in stimulating the crayfish immune system, and the lower THC on day 14 was determined by the acceptance of FPKM in crayfish.

The hemocyte increase showed that the crayfish tried to preserve homeostasis by producing more blood cells. According to Chen *et al*. [1], increased hemocyte cell counts are induced by increased cardiac contractility in shrimp owing to a homeostatic response induced by changing environmental factors. In this study, the number of hematocytes reached 10^5^ cells/mL. This number is below the normal range for hematocytes in crustaceans. According to Achmad *et al*. [25], the normal THC in marron (*Cherax cainii*) of 7.14 ± 2.08 g is 1.25 × 10^6^ cells/mL. The hemocytes of crustaceans are involved in phagocytosis, encapsulation, nodule formation, injury healing, blood coagulation, and proPO activation [26].

Based on the ANOVA results, there was no significant effect (p > 0.05) on hyaline or granular cells. Based on these observations, hyaline cells have a smooth shape, with no or few granules. According to Duangsuwan *et al*. [27], hyaline cells are the smallest cell type, with a high nuclear-cytoplasmic ratio and no or few granular cytoplasm. The percentage of hyaline cells in this study ranged from 41% to 57%, which is in a normal range; this is confirmed by Saputra *et al*. [28], the normal value of hyaline cells in *Cherax cainii* ranged from 50% to 71%. Granular cell observations revealed that the cells were round and filled with multiple granules. According to Sayyaf Dezfuli *et al*. [29], granular cells are the largest cell type, with smaller nuclei covered with granules. The percentage of granular cells observed in this study ranged from 42% to 58%, which is within the normal range. According to Zhang *et al*. [2], the number of granular cells in normal crayfish varies between 51% and 62%. Therefore, each hematocyte cell has a different function. Hyaline cells are useful for phagocytosis, and granule cells are useful for the encapsulation, storage, and release of proPO and cytotoxicity [30].

In this study, PA increased after treatment. The highest value was observed for treatment P3 on day 7, at 74%. The PA results show initial stability across all treatment groups, followed by a notable increase on days 5, 7, and 14, particularly in P2 and P3. This increase suggests that FPKM supplementation enhances immune function by stimulating phagocytic cell activity.

The observed trend can be explained biologically by bioactive compounds in FPKM, such as short-chain peptides and beneficial microbial metabolites, which may enhance immune cell activation. Furthermore, fermentation enhances nutrient bioavailability, thereby improving immune system function. The significant increase in PA on days 7 and 14 suggests a delayed but sustained immune response, indicating the long-term benefits of FPKM in strengthening nonspecific immunity. The increased PA showed a rising immune defense response in the crayfish, which responds to invading organisms through the phagocytosis process [31]. Shi *et al*. [32] reported that immunostimulants can stimulate hemocyte cells to degranulate so that proteins are released and increase the number of phagocytic cells. In measuring the PI value, the results of the ANOVA test showed a significantly different effect (p > 0.05) on days 5 and 7. Based on the results of the increase in the PI value, the ability of phagocyte cells to digest extraneous particles was higher than that in the control treatment or pretreatment period.

The next step was to monitor the PO’s activity. This enzyme is important for the immunity of crustaceans because it promotes the production of melanin, which responds to extraneous organisms inside the crayfish. In this study, the PO value increased after treatment. The highest PO value occurred on day 5 when FPKM was supplemented at up to 120 g/kg of feed. The results of the ANOVA test showed that the application of FPKM to the feed increased the PO value (p < 0.05). The prebiotic content of FPKM, namely MOS, can increase proPO activity. Several previous studies have reported the effect of prebiotics on increasing proPO activity. The proPO enzyme is an inactive form of PO. The transformation of proPO into PO involves several reactions known as the proPO activating system. The application of MOS in *L. vannamei* can increase PO activity [33]; in *Procambarus clarkii*, proPO increased significantly with the administration of 10 mg/g FOS [34], and in *L. vannamei*, proPO activity increased after being administered inulin as much as 2.5 mg/g [35]. Increased PO activity improves the ability of crayfish to recognize foreign particles in the body and can increase the phagocytosis process. This result was demonstrated by the increase in PA observed in this study.

The activity of SODs was also analyzed. It is the primary antioxidant that helps prevent reactive oxygen species (ROS) and induces oxidative stress [36]. The best results were obtained using 120 g/kg of FPKM in the feed on day 14. The increase in SOD activity is attributed to the MOS content in FPKM, which stimulates the release of SOD to suppress excess ROS in crayfish. This finding follows the assertions of Liu *et al*. [37] that high-fiber foods have prebiotic and antioxidant activities against radicals. MOS has a hydroxyl group that can react with superoxide anions, hydrogen peroxide, and hydroxyl radicals [38]. MOS can also increase the concentration of anti-superoxide anion (ASA) and anti-hydroxyl radical (AHR). The increased ASA and AHR values indicate that crayfish tissues can inhibit superoxide anions and radicals [38].

The fermentation of PKM using *A. niger* introduces a novel approach to enhancing prebiotic efficacy. The stability of the TPP values observed in this study may reflect the unique biochemical properties of FPKM, suggesting a new avenue for immune modulation in crustaceans. Various factors influence TPP levels in crustaceans, including molting, reproduction, nutrition, infection, hypoxia, and salinity [39]. The amount of total protein in the plasma is also species-dependent; for example, the total protein in shrimp is higher than that in crayfish [38]; Paul *et al*. [40], Nababan *et al*. [41] reported that TPP can fluctuate in response to infection by the disease. An increase in TPP implies that the crustacean is infected, whereas a decrease in TPP implies stress or poor nutrition.

The expression of lectin genes was significantly upregulated in this study, although several treatments were downregulated. The expression of the lectin gene was also higher than that of the LGBP gene. Treatment with FPKM at 80 g/kg feed showed no decrease in gene expression, with the highest upregulation on day 7 at 187.9 times. Treatment with FPKM at 40 g/kg feed showed the most significant increase in expression (52,299.2 times compared with the previous day, which was down-regulated. Treatment with FPKM at 120 g/kg feed showed a more stable value, with the highest expression on day 14 at 27,609.9 times. LGBP gene expression showed lower results compared to the lectin gene. Treatment with FPKM at 40 g/kg feed showed the highest upregulation on day 14. Treatment with FPKM at 40 g/kg feed also showed the best upregulation. An increase in immune gene expression can be caused by the provision of immunostimulants, such as prebiotics or probiotics [16, 21, 33, 41, 42].

The increased gene expression observed in this study suggests that the MOS structure in FPKM can bind lectin in hemocytes, facilitating immune activation. LGBP plays a crucial role in recognizing LPS and β-glucans in bacterial cell walls. Upon antigen recognition, LGBP triggers hemocyte degranulation and stimulates the proPO system, a key component of the crustacean immune response. In addition, LGBP-mediated immune activation enhances essential defense mechanisms such as phagocytosis, opsonization, and agglutination, thereby strengthening hemocyte function against pathogens [33].

## CONCLUSION

The study conclusively demonstrates that dietary supplementation with FPKM effectively enhances non-specific immune responses in Indonesian red claw crayfish (*C. quadricarinatus*). Significant improvements were observed in immune parameters, including increased THC, elevated phagocytic activity (PA), and PI, as well as heightened activities of SOD and PO. Optimal immunological responses were most consistently recorded at the dietary inclusion level of 120 g/kg FPKM. In addition, the molecular analysis revealed notable upregulation of immune-related genes such as lectin and LGBP, underscoring the immune-stimulating properties of FPKM-derived prebiotics.

The primary strength of this study lies in its comprehensive integration of physiological and molecular assays to evaluate immune responses, providing robust evidence for the immunomodulatory potential of FPKM in crustacean aquaculture. In addition, employing a locally sourced agricultural by-product promotes sustainable and environmentally friendly aquaculture practices, reducing dependence on synthetic antibiotics.

However, limitations include the relatively short duration of the study and the focus exclusively on non-specific immune parameters without assessing direct pathogen resistance *in vivo*. Furthermore, variability in individual crayfish responses highlights the need for larger-scale trials.

Future research should involve extended trial durations and field-based studies to validate laboratory findings under commercial aquaculture conditions. Further investigation into pathogen challenge experiments would be beneficial to directly correlate immune enhancement with disease resistance. Expanding research to explore synergistic effects between FPKM and other probiotic strains or dietary supplements could also provide valuable insights for the sustainable advancement of crayfish aquaculture.

## AUTHORS’ CONTRIBUTIONS

DDTW: Conceptualized and designed the study. HNF, MWY, and PCD: Sample collection and laboratory analysis. YE, AS, HPF, MKA, and AW: Data analysis and interpretation and manuscript preparation. MKA: Drafted the manuscript. All authors actively contributed to the revision of the manuscript and approved the final manuscript.
